# Is Adding HCV Screening to the Antenatal National Screening Program in Amsterdam, The Netherlands, Cost-Effective?

**DOI:** 10.1371/journal.pone.0070319

**Published:** 2013-08-12

**Authors:** Anouk T. Urbanus, Marjolijn van Keep, Amy A. Matser, Mark H. Rozenbaum, Christine J. Weegink, Anneke van den Hoek, Maria Prins, Maarten J. Postma

**Affiliations:** 1 Cluster of Infectious Diseases, Public Health Service, Amsterdam, The Netherlands; 2 Center for Infection and Immunology Amsterdam (CINIMA), Academic Medical Centre (University of Amsterdam), Amsterdam, The Netherlands; 3 Department of Pharmacy, Unit of PharmacoEpidemiology & PharmacoEconomics (PE2), University of Groningen, Groningen, The Netherlands; 4 Julius Center for Health Science and Primary Health Care, University Medical Center Utrecht, The Netherlands; 5 Department of Gastroenterology and Hepatology (AMC Liver Centre), Academic Medical Centre (University of Amsterdam), Amsterdam, The Netherlands; University of North Carolina School of Medicine, United States of America

## Abstract

**Introduction:**

Hepatitis C virus (HCV) infection can lead to severe liver disease. Pregnant women are already routinely screened for several infectious diseases, but not yet for HCV infection. Here we examine whether adding HCV screening to routine screening is cost-effective.

**Methods:**

To estimate the cost-effectiveness of implementing HCV screening of all pregnant women and HCV screening of first-generation non-Western pregnant women as compared to no screening, we developed a Markov model. For the parameters of the model, we used prevalence data from pregnant women retrospectively tested for HCV in Amsterdam, the Netherlands, and from literature sources. In addition, we estimated the effect of possible treatment improvement in the future.

**Results:**

The incremental costs per woman screened was €41 and 0.0008 life-years were gained. The incremental cost-effectiveness ratio (ICER) was €52,473 which is above the cost-effectiveness threshold of €50,000. For screening first-generation non-Western migrants, the ICER was €47,113. Best-case analysis for both scenarios showed ICERs of respectively €19,505 and €17,533. We estimated that if costs per treatment were to decline to €3,750 (a reduction in price of €31,000), screening all pregnant women would be cost-effective.

**Conclusions:**

Currently, adding HCV screening to the already existing screening program for pregnant women is not cost-effective for women in general. However, adding HCV screening for first-generation non-Western women shows a modest cost-effective outcome. Yet, best case analysis shows potentials for an ICER below €20,000 per life-year gained. Treatment options will improve further in the coming years, enhancing cost-effectiveness even more.

## Introduction

Hepatitis C virus (HCV) is primarily a blood-borne virus and causes persistent viremia in about 75% of those infected [1]. Over the course of decades, chronic HCV infection can lead to liver cirrhosis and, eventually, death. HCV infection is an asymptomatic disease and as such, treatment is mostly initiated in an advanced stage of disease [1].

In high-income countries, health-care associated HCV transmission was effectively halted by the introduction of donor blood screening in 1991. As a result, the vast majority of new HCV infections occur among specific risk groups, in particular injecting drug users (IDUs) through sharing of injection equipment [2]. In contrast, in low- and middle-income countries, the majority of HCV transmissions remains health-care associated primarily due to inadequately sterilized syringes and medical equipment [1].

In the Netherlands, HCV prevalence is estimated at 0.22% (min: 0.07% max: 0.37%) [3]. Blood donors and HIV positives are routinely screened for HCV, but there is no universal screening policy for HCV that targets the general population. In the past decade, several national and regional HCV (pilot) screening campaigns have been conducted in the Netherlands for specific risk groups, such as active drug users participating in harm-reduction programmes [4], [5] as well as others hidden in the general population (e.g. those who have had a blood transfusion or injected drugs one time in the remote past). The latter campaign only ran for limited periods of time [4], [6].

A recent study in the Netherlands showed HCV prevalence among indigenous pregnant women of 0.26% (95% CI: 0.15–0.46), which is similar to the prevalence in the general population [7]. However, the prevalence was somewhat higher among first-generation migrants from non-Western countries (0.70%; 95% CI: 0.43–1.29) [7]. The transmission rate from mother to child is estimated to be around 5% in HIV-negative mothers, depending on the viral RNA load of the mother [8].

Currently, HCV-infected patients are treated with a weekly pegylated interferon injection plus a daily oral dose of ribavirin. Genotypes 1 and 4 are more difficult to treat than genotypes 2 and 3. Two protease inhibitors (boceprevir and telaprevir) have been recently licensed for treatment of HCV infection with genotype 1 in the Netherlands. Although these new treatment options are more expensive, when added to pegylated interferon and ribavirin, the response rate improves substantially [9], [10]. With even more effective treatment to be expected, it becomes increasingly important to identify undiagnosed HCV-infected individuals. Identifying HCV-infected individuals can lighten the future burden of disease and help prevent secondary transmission.

HCV screening programmes in populations with low HCV prevalence and standard treatment are mostly not cost-effective [4], [11], [12]. The reasons for this are the relatively low prevalence and treatment outcome, screening setting and discount rate and the willingness –to -pay of the public, which depends on several economic, social and political factors [11]. Yet, HCV screening in settings where screening for other infectious diseases already exists might be cost-effective. In the Netherlands, as in many other countries, pregnant women are regularly screened in the third month of pregnancy for several infectious diseases, including hepatitis B virus (HBV) and HIV [13]. Therefore, adding HCV testing to this screening procedure will only require a minor adjustment, limited investment, and low costs. Both the HCV-infected mother and her child would benefit from prenatal screening, because treatment can start relatively early in the course of infection and thus avert serious HCV-related complications. To examine whether adding HCV testing to routine screening for pregnant women is cost-effective, we developed a Markov model, taking the benefits for the mother into account. We used HCV-prevalence data among pregnant women collected in 2003, including ethnicity. Scenario studies were done to estimate whether implementation of HCV screening for all pregnant women was cost-effective and whether it was cost-effective to screen only first-generation non-Western women. In addition, cost-effectiveness of various treatment scenarios was explored.

## Methods

### Ethics statement

The medical ethics committee of the Academic Medical Centre (MEC AMC) approved the current study.

**Figure 1:**
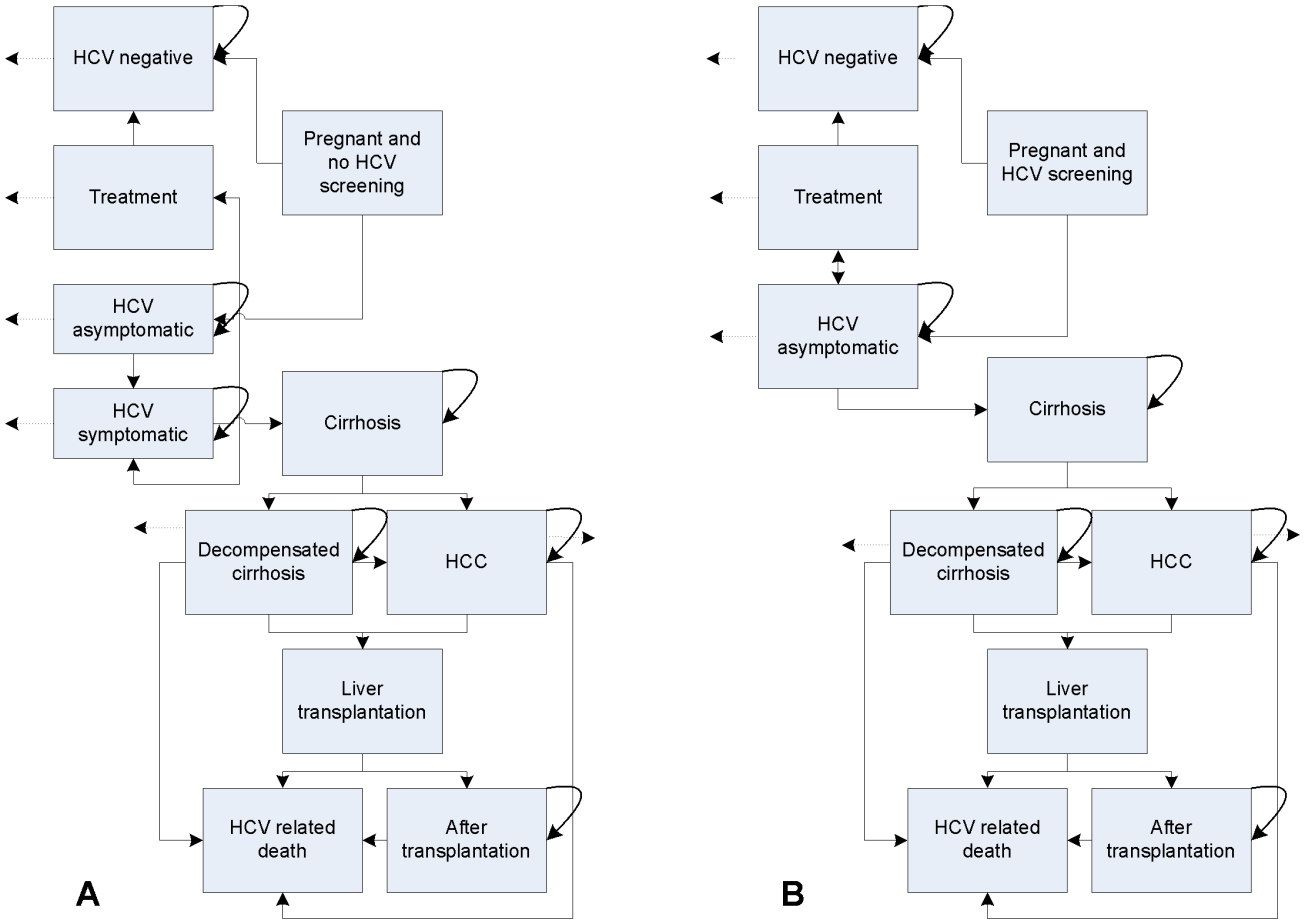
Schematic description of the Markov model. Annually, women move between health stages according to defined transition rates given in Table 1. The natural history of HCV infection (hepatitis C virus) is modelled through the stages of chronic infection, cirrhosis, decompensated cirrhosis, hepatocellular carcinoma (HCC), liver transplantation, and the years after transplantation. The dotted arrows indicate competing mortality. In Figure 1A the model is presented for the women who are not routinely screened for HCV during their pregnancy and are diagnosed in a later stage of infection, in Figure 1b the model is presented for women who are routinely screened during their pregnancy.

In this analysis, we express cost-effectiveness as the ratio of the net expenditures and net health outcomes. The net expenditures reflect the difference in costs between a situation where screening is conducted and a situation where there is no routine screening, reflecting current practice. Screening is deemed cost-saving if costs in the screening scenario are lower than current practice where no screening is conducted. If net expenditures are positive, sufficient positive health gains are needed to make the screening cost-effective. In the Netherlands, screening is certainly deemed to be cost-effective if the cost per life-year gained (LYG) is ≤€20,000, and potentially still cost-effective up to €50,000 per LYG [14]. The incremental cost-effectiveness ratio (ICER) is found by:
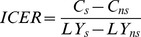
where *C* are the costs and *LY* are life-years in the scenario with screening (*s*) and without screening (*ns*), respectively. The net costs and net LYG are calculated as the difference in total costs and LYG with and without screening. Each year a woman is alive in the Markov model (see Figure 1) is counted as a life-year, independent of the transition state she is in. This analysis is conducted from a health care perspective, only accounting for direct medical costs.

We expressed all costs in 2011 price levels and discounted them at an annual rate of 4%. Life-years were discounted at an annual rate of 1.5%, according to Dutch guidelines [8]. The model was built using Microsoft Excel 2007.

### Markov model

We developed a Markov model to compare current practice (no routine HCV screening) with HCV screening of women during pregnancy and subsequent treatment (scenario 1). We assumed that women who receive no routine HCV screening will be screened by a GP when developing symptoms in a later stage of chronic infection. Women were eligible to enter the model at pregnancy at 31 years of age and for first-generation non-Western women at 29 years of age, approximating the average age of first pregnancy in the Amsterdam region [15]. Women at entrance did not have HCV symptoms and were HIV-negative, because we assumed that indeed otherwise they would have been diagnosed already. It was assumed that 42% of the anti-HCV positive women had already cleared the infection spontaneously [16]. In the model, these women were considered HCV-negative.

In the current practice scenario (scenario 1), women were either HCV negative or HCV asymptomatic at model entrance. From the HCV-asymptomatic stage, they moved through the different optional HCV health stages (e.g., symptomatic infection, cirrhosis, decompensated cirrhosis, hepatocellular carcinoma (HCC), and liver transplantation) (Figure 1A), according to annual transition probabilities given in Table 1. Once diagnosed with HCV during pregnancy, we assumed that 50% of the diagnosed women received treatment in the second year after diagnosis. In this scenario, treatment consisted of peginterferon alfa and ribavirine for genotype 2–4 with added protease inhibitors for genotype 1. We refer to this as ‘new treatment regimen’. It was assumed that cirrhosis does not develop during HCV therapy. If therapy was not successful, women remained chronically infected and return to the (a)symptomatic stage. After liver transplantation, women moved to the ‘after transplantation’ state until death. The model included two types of mortality: competing mortality (due to causes unrelated to HCV) and HCV-related mortality (through decompensated cirrhosis, hepatocellular carcinoma [HCC] or liver transplantation). The probabilities of competing deaths and liver cirrhosis were age-dependent. In this scenario (scenario 1), we assumed that asymptomatic infections were not diagnosed; there were no costs related to this health stage and there was no treatment possibility. Medical costs were counted for all symptomatic HCV stages.

**Table 1:** Overview of annual transition probabilities and cost variables used in the Markov model.

Variable	Value	Distribution/range	Reference
Probability of HCV infection, all women	0.002#	Beta (9, 4555)	[15]
Probability of HCV infection, first generation non-Western migrants	0.0043#	Beta (7, 1605)	[15]
Transition from asymptomatic to symptomatic HCV	0.012		[4]
Percentage genotype 1*	24%		[15]
Percentage genotype 2	22%		
Percentage genotype 3	30%		
Percentage genotype 4	24%		
Transition from chronic disease to treatment	0.50		[4], [25]
Probability of successful treatment outcome:			
*Scenario 1 (new protease inhibitors)* **			
Genotype 1	0.70		[26]
Genotype 2 and 3	0.78		[27]
Genotype 4	0.56		[27]
*Scenario 2 (standard of care)*			
Genotype 1	0.40		
Genotype 2 and 3	0.78		
Genotype 4	0.56		
*Scenario 3 (possible future regimen)*			
Genotype 1	0.78		
Genotype 2 and 3	0.78		
Genotype 4	0.78		
Transition to cirrhosis per year$		Range:	[18]
20–39 years	0.000	0.00–0.001	
40–49 years	0.001	0.00–0.002	
50–59 years	0.004	0.003–0.005	
60–69 years	0.005	0.003–0.007	
>70 years	0.019	0.015–0.02	
Transition from cirrhosis to decompensated cirrhosis	0.039	Beta: (14.617, 260.1732)	[27]
Transition from cirrhosis to HCC	0.015		[12]
Transition from decompensated cirrhosis to HCC	0.015		[12]
Transition from decompensated cirrhosis to liver transplantation	0.031		[12]
Transition from decompensated cirrhosis to HCV related death	0.129		[12]
Transition from HCC to liver transplantation	0.031		[12]
Transition from HCC to HCV-related death	0.43	Beta (117.1–155.23)	[27]
Transition from post transplantation to HCV-related death	0.21	Beta (430, 1617).	[12], [28]
Transition after transplantation to HCV-related death	0.057	Beta (112, 2027)	[12], [28]
Competing mortality		Depending on age	[29]
**Costs**			
Cost, antibody HCV test	€12.69		Based on PHSA Laboratory prices
Cost, RNA-test	€122.11		Based on PHSA Laboratory prices
Cost, chronic infection, per year	€158.73	Range: €79.37–€317.46	[23]
Cost treatment			
*Scenario 1 (new protease inhibitors)* **			
Genotype 1 (24 w)	€34,900	(Mean cost for Boceprevir and telaprevir)	[30]
Genotype 2/3	€9830		[31]
Genotype 4	€16,178		[31]
*Scenario 2 (standard of care)*			
Genotype 1	€16,178		
Genotype 2/3	€9,830		
Genotype 4	€16,178		
*Scenario 3 (possible future regimen)*			
Genotype 1	€34,900		
Genotype 2/3	€34,900		
Genotype 4	€34,900		
Annual cost cirrhosis	€821.73	Range min - max: €410.87–€ 1,643.47	[23]
Cost decompensated cirrhosis, per year	€27,921.72		[32]
Cost HCC, per year	€21,054.92	Range: €10,527.46–€42,109.85	[23]
Cost liver transplantation	€143,226.96	Range: €71,613.48–€286,453.93	[23]
Cost after transplantation, per year	€20,714.27	Range: €10,357.13–€41,428.53	[23]

HCV: hepatitis C virus.

HCC: hepatocellular carcinoma.

# In the prevalence a clearance rate of 42% [14] was included. The prevalence used in the model for all pregnant women is 0.2% (9/4563; 95% CI: 0.10–0.37) and for first generation non-Western women 0.43% (7/1612; 95% CI 0.21–0.89).

$ Transition rate is age-dependent.

* same distribution was used for first-generation non-Western women.

** new protease inhibitors are added to the standard of care regimen (peginterferon alfa and ribavirine).

In the base-case analysis (scenario 1a), women entered the model and were screened for HCV. Women moved to either the HCV-negative or the chronic-HCV infection state. It was assumed that women who were screened and diagnosed were chronically infected with HCV, and thus HCV progression was modelled as in the current practice scenario (Figure 1B). Treatment was administered according to the new treatment regimen.

The same situation was estimated for only first-generation non-Western pregnant women (scenario 1b), because non-Western countries in general have a higher HCV prevalence [7]. Western was defined as Western Europe (excluding Portugal, Spain, and Italy because of higher HCV rates), Australia, New Zealand, and North America; all other countries were categorized as non-Western. Ethnicity was determined by the country of birth of the woman's mother. If the mother was native Dutch, ethnicity was determined by the birth country of the participant's father.

In scenario 2a (screening all women) and scenario 2b (screening only first-generation non-Western women), all women diagnosed with HCV were treated with peginterferon alfa and ribavirine, the standard of care regimen. In scenarios 3a (screening all women) and scenario 3b (screening only first generation non-Western women), we used a hypothetical future treatment regimen where the new protease inhibitors were added to the standard of care treatment for all genotypes.

### Analysis

To account for uncertainty, beta distributions were used for the transition-probability parameters [17]. All variables, including distributions and ranges are summarized in Table 1. We performed 10,000 simulations. For every run, a set of parameters was sampled from the parameter space. For parameters where the 95% confidence interval was not available, a range of 20% around the point estimate was used as the standard deviation. For the cost parameters, the standard deviation was assumed to be as high as the mean cost [17], assuming a gamma distribution.

### One-way sensitivity analysis

A sensitivity analysis was performed to examine the contribution of the various parameters to variation in ICERs, represented in a tornado diagram. To obtain the respective ranges each parameter was increased or reduced once a time with 25%.

In addition, we performed a best-cases analysis for both scenarios, with all parameters in the model plus or minus 25% to optimize cost-effectiveness. Finally, we estimated to what extent treatment costs should decline in order for HCV screening to conform to a cost-effective threshold of €20,000.

### Epidemiological aspects

We used data from the 2003 routine screening, in which all pregnant women in their 10^th^ to12^th^ week of pregnancy were routinely tested for hepatitis B, syphilis and HIV at the local antenatal clinics in the Amsterdam area. The screening from 2003 was chosen for retrospective HCV testing because ethnicity data were collected. Annually, 10,000 to −13,000 women are tested. Further details on this dataset are described elsewhere [13], [15]. As noted, women were tested retrospectively by means of an HCV-antibody test, and positive test results were confirmed with an immunoblot. Positive antibody test results were then tested for HCV RNA and genotyped.

### Costing aspects

The costs for the different health states were derived from the literature and indexed to 2011 prices (Table 1). Our analysis included the cost of HCV screening, medication (including pharmacists' fees), diagnostic tests, costs for liver transplantation, as well as for decompensated cirrhosis and HCC.

## Results

### Base-case analysis

The incremental cost per woman screened was €41, and 0.0008 life-years were gained in the scenario in which all pregnant women were screened, resulting in an ICER of €52,473. For screening only first-generation non-Western women, the ICER was €47,113 (see Table 2). Screening only pregnant women that migrated from non-Western countries was more cost-effective than screening all pregnant women. In both scenarios, the ICER was above the certain cost-effectiveness threshold of €20,000, but the ICER for first-generation non-Western migrants was under the €50,000 threshold and therefore moderately cost-effective.

**Table 2:** Cost-effectiveness outcomes for all pregnant women and first-generation non-Western women (scenarios 1a and 1b), based on probabilistic uncertainty analysis (10000 simulations).

		Mean costs	Mean life years	Incremental costs	LYG	ICER (€/LYG)
All pregnant women	Screening	€ 55,474	35492.8	€ 41,869	0,80	€ 52,473
	No routine screening	€13,605	35492.0			
non-Western migrants	Screening	€106,307	36378.6	€ 77,582	1,65	€ 47,113
	No routine screening	€ 28,725	36377.0			

The incremental cost-effectiveness ratio (ICER) is calculated with reference to the “no routine screening” strategy.

LYG:life years gained.

ICER: incremental cost-effectiveness ratio.

### Best-case scenario

Table 3 shows the results of the best-case scenarios. When screening all women or only first generation non-Western pregnant migrants, the ICER in the best-case scenario is below the potential cost-effectiveness threshold of €20,000, with €19,505 for all pregnant women and €17,533 for first-generation non-Western women.

**Table 3:** Best-case scenarios for screening all pregnant women (scenario 1a) and first-generation non-Western women (scenario 1b).

		Mean costs	Mean life years	Incremental costs	LYG	ICER (€/LYG)
All pregnant women	Screening	€ 41,809	35492.8	€ 30,228	1,55	€ 19,505
	No routine screening	€ 11,581	35491.3			
non-Western migrants	Screening	€ 78,978	36378.6	€ 55.320	3,16	€ 17,533
	No routine screening	€ 23,658	36375.4			

With parameter optimization ±25% and incremental cost-effectiveness ratio calculated with reference to the “no routine screening” strategy.

LYG:life years gained.

ICER: incremental cost-effectiveness ratio.

We estimated that if treatment costs decline to €3,750, screening pregnant women will be cost-effective at a threshold of €20,000. A decline of treatment costs to €6,750 for first generation non-Western women will also be cost-effective.

### Treatment scenarios

We examined a scenario in which standard of care treatment was given to all women infected with genotypes 1–4 (scenarios 2a and 2b). In both scenarios, adding HCV screening to an already existing routine screening program was probably cost-effective. Screening all pregnant women resulted in an ICER of €44,952, and screening only first-generation non-Western women resulted in an ICER of €38,861. Best-case scenarios revealed ICERs of €16,313 and €14,153, respectively, for screening all women and for non-Western migrants only. A decline of treatment costs to €3,500 and €6,000 respectively will be cost-effective at a cost-effective threshold of €20,000.

In scenario 3, in which all women with genotype 1–4 would be on the new improved treatment regimen, HCV screening was not cost-effective with an ICER of €88,162. The same was true for non-Western women with an ICER of €86.005. Best-case scenarios revealed ICERs of € 42,270, and € 32,853, respectively, for screening all women and for screening non-Western migrants only. A decline of treatment costs to €4,250 and €7,250 respectively will conform to the cost-effective threshold of €20,000.

### Sensitivity analysis

**Figure 2:**
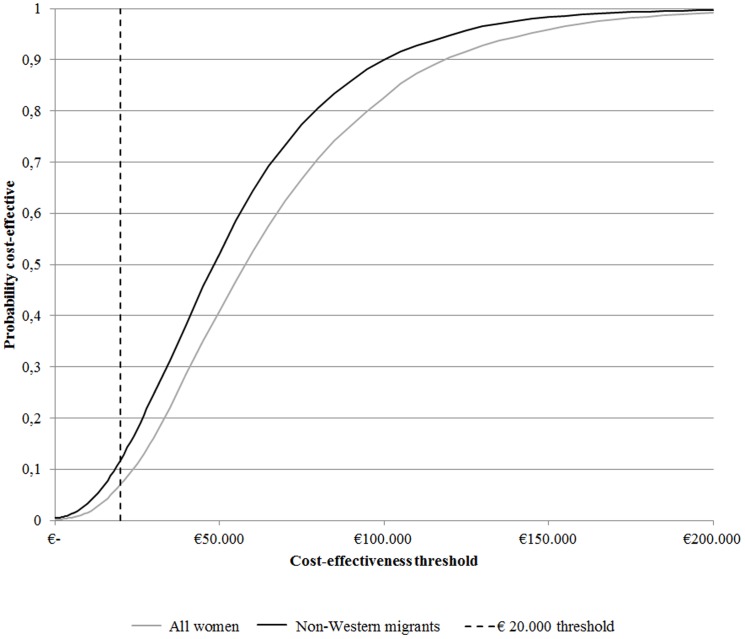
Cost-effectiveness acceptability curves (CEAC) for all pregnant women (indicated with the black line) and for first-generation non-Western women (indicated with the grey line) for scenarios 1a and 1b. The graph shows the probability of screening being cost-effective when different cost-effectiveness thresholds are used, resulting from uncertainty analysis. In the Netherlands, the certain cost-effective threshold is €20,000 (indicated by the dotted line) and regimens that are calculated at €20,000 and €50,000 are potentially cost-effective. As shown in both scenarios, 10% of the simulations were below the cost-effectiveness threshold of €20 000.

The cost-effectiveness acceptability curves (CEACs) derived from the sensitivity analysis are given in Figure 2, which shows that screening all pregnant women is probably not cost-effective. However, screening only first-generation non-Western migrants is probably cost-effective.

**Figure 3:**
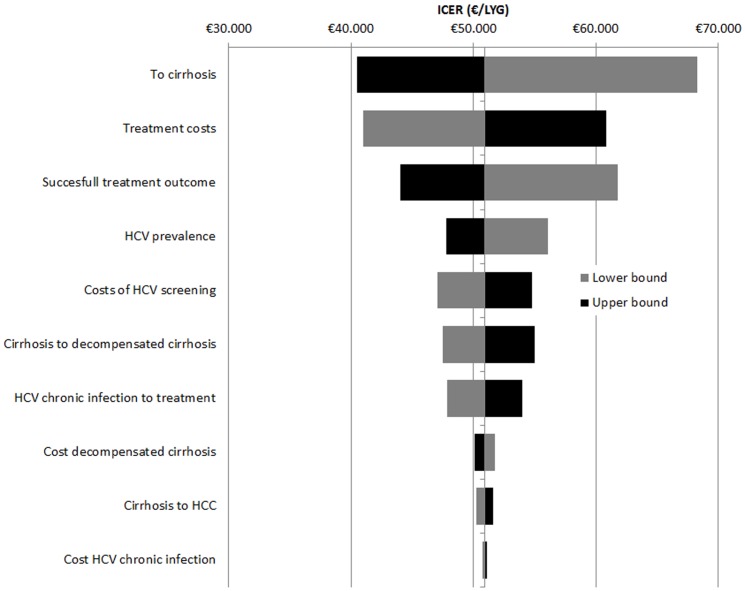
Tornado diagrams of the sensitivity analyses. Diagram A) describes scenario 1a and diagram B) scenario describes scenario 1b. Both diagrams show the change in ICER when reducing or increasing each parameter with 25%.

The one-way sensitivity analysis showed that the ICER for screening all pregnant women is most sensitive to changes in the transition probabilities to cirrhosis, as shown in the tornado diagram in Figure 3a. Other parameters that have a large impact on the outcome are the treatment costs, successful treatment outcome, the prevalence of HCV, the costs of HCV testing, the transition from cirrhosis to decompensated cirrhosis, and the probability from chronic HCV infection to treatment. The sensitivity analysis for first generation non-Western women showed also that the ICER for screening only this specific group of women is most sensitive to changes in the probability rates of transition to to cirrhosis (see Figure 3b). Other parameters that have a large impact on the outcome are the same as discussed above.

In addition we performed our analysis with different discount rates. If a discount rate 0% for both costs and life years was used the ICER for respectively all pregnant women and first generation non-Western women was €10,745 and €7,099. Discounting at 3% we found an ICER of respectively €98,637 and €90,818 and discounting at 4% we found an ICER of respectively € 166,494 and €157,633..

## Discussion

In this study, we found that screening all pregnant women in Amsterdam for HCV within the existing screening program for other infections during pregnancy is probably not cost-effective. Screening first-generation non-Western women was moderately cost-effective. These findings are partly due to the slow progression of HCV infection to cirrhosis, especially for women [18], and the relatively high costs for patients treated with new protease inhibitors (boceprevir and telaprevir). However, standard of care treatment is estimated in the literature to be cost-effective in treatment-naïve patients [19]. Therefore we also estimated the ICER when standard of care treatment is used (scenarios 2a and 2b) and found a more favourable ICER than when new treatment options are used, but still above the certain cost-effective threshold of €20,000. Other studies found that HCV screening in relatively low-prevalence or low-risk populations is not cost-effective [12], [20]. One study indicated that screening in a migrant population is only cost-effective if the HCV prevalence is at least 2% [21]. Initially, we assumed that when HCV screening is integrated into an existing screening program it may be cost-effective, since expenditures for extending an already existing screening are low and the only costs are for testing.

Notably, the low prevalence found in our study population -which means that high numbers are required for screening detect one infected person- was another reason for the slightly unfavourable cost-effectiveness in all pregnant women.

The best-case analyses show the potential for an ICER below the threshold of €20,000 per life-year gained. In a separate analysis, we also found a favourable cost-effectiveness for the standard of care treatment (scenarios 2a and 2b). However, we did not find a favourable cost-effectiveness in the best-case analysis of scenario 3a and 3b, where all women, independent of the genotype, were on the new treatment regimen. The high cost of the new hypothetical future treatment regime obviously influenced the cost-effectiveness outcome negatively.

We included new treatment options for genotype 1, which were licensed in 2012. Although treatment outcomes have improved with these new medications, costs are still high because of patents on the medications. We estimated that if treatment costs decline to €3,750 per treatment, screening pregnant women will be cost-effective. Unfortunately, it is unlikely that any decrease in costs of the newly approved drugs will be large enough (this involves a reduction in price of €31,000) to result in favourable cost-effectiveness in the upcoming years. However, potent treatment options, without peginterferon, are expected to be available with shorter treatment durations, fewer side-effects [22] and with more favourable costs in the future. The probability of receiving treatment will increase as well, because of the better treatment options and thus result in a more favourable cost-effectiveness ICER.

In addition, we might have overestimated the duration and the costs of treatment and underestimated the ICER because in practice women can receive a shortened treatment because they have achieved a rapid virological response in the early phase of treatment.

In this study only direct medical costs and benefits are included; indirect costs were not considered. Since side effect could be very severe this could influence the outcome negatively. Some costs were derived from literature [23], and converted to 2011 index prices. Also, we only measured life-years and did not take other health outcomes into account, which means that a life-year spent with diseases contributes as much to the ICER as a healthy life-year. Using health-related quality of life next to life-years would probably give a more cost-effective outcome, because screening may detect HCV-infected women years earlier and thus prevent years of discomfort and severe complications in a later stage of life. However, women can also experience a small loss of quality of life because of early detection during their pregnancy. Treatment is a contraindication during pregnancy and the small chance of mother-to-child transmission can therefore not be averted which could give the mother stress. Nevertheless, we believe that more quality is gained than lost by early detection. In order to test our assumptions, quality of life will be included in our model in a following study on pregnancy and HCV.

We ignored the costs of health care and screening in the child because the transmission rate from mother to child is relatively low at 5% (depending on the RNA load of the mother); also, the prevalence in mothers is low [8], and therefore the costs related to the mother and her infection vastly outweighs the small costs for the children. Taking the benefits for the child into account possibly has a small effect on the ICER. When the mother is diagnosed with HCV, the child can be closely monitored for possible transmission with HCV and start treatment when indicated. Unfortunately, there are limited options to prevent mother-to-child transmission, since the precise transmission route from mother to child is unknown, most likely transmission occurs during birth [8]. Treatment of pregnant women is not indicated and cannot prevent transmission to the child, because neither birth by caesarean section nor lack of breastfeeding lower the transmission rate significantly [8]. Because of the transmission rate, HCV screening during pregnancy is probably not cost-effective for the child either.

Although implementing HCV screening during pregnancy in an existing routine screening program for infectious diseases with the currently available treatment regimen may not be cost-effective for all pregnant women, the ICER for first-generation non-Western women shows a modest cost-effectiveness outcome. In line with other studies [11], our results suggest that risk based screening could be cost-effective in low prevalence counties like the Netherlands. Since first-generation non-Western migrants comprise a large proportion of the undiagnosed HCV-infected population in the Netherlands, this risk group should be targeted for screening [3]. This, together with the high screening uptake of 99.8% [24] in the existing routine should argue for implementation of HCV screening for first-generation non-Western women. In addition, the best-case analysis shows potential for an ICER below the €20,000 per LYG. Furthermore, more improved treatment outcomes, without peginterferon and with shorter treatment duration, are expected in the coming years, which are likely to enhance cost-effectiveness even more.
